# Supplementary material for: Long-term depression in neurons involves temporal and ultra-structural dynamics of phosphatidylinositol-4,5-bisphosphate relying on PIP5K, PTEN and PLC
Source: Commun Biol. 2023 Apr 3;6:366. doi: 10.1038/s42003-023-04726-0 (PMC10070498; doi:10.1038/s42003-023-04726-0)
Supplement: Supplementary file 2 — Supplementary Information [file 42003_2023_4726_MOESM2_ESM.pdf]

Supplementary Information

Long-term depression in neurons involves temporal and ultra-structural dynamics of phosphatidylinositol-4,5-bisphosphate relying on PIP5K, PTEN and PLC

Sarah A. Hofbrucker-MacKenzie[1], Eric Seemann[1], Martin Westermann[2], Britta Qualmann[1*], Michael M. Kessels[1*]

[1] Institute of Biochemistry I, Jena University Hospital – Friedrich Schiller University Jena, 07743 Jena, Germany

[2] Center for Electron Microscopy, Jena University Hospital – Friedrich Schiller University Jena, 07743 Jena, Germany

* Correspondence

Britta.Qualmann@med.uni-jena.de and Michael.Kessels@med.uni-jena.de

Supplementary Figure 1

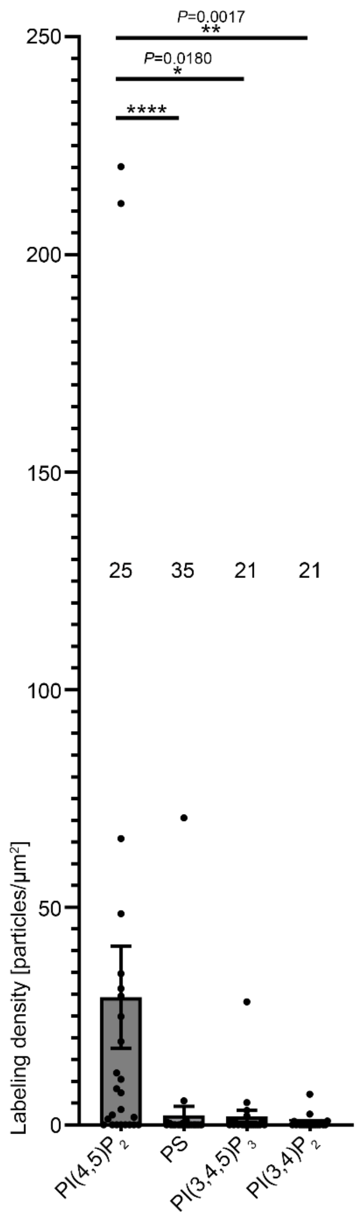

Supplementary Figure 1. Anti-PIP_2 antibodies specifically recognize PIP_2 at freeze-fractured liposomes

Quantitative analyses of labeling densities, which were presented in **Fig. 1e** as bar plots, as bar plots with all individual data points showing the typical wide spread of labeling densities in quantitative TEM analyses of immunogold labelings. Data, mean±SEM. PI(4,5)P_2, n=25; PS, n=35; PI(3,4,5)P_3, n=21; PI(3,4)P_2, n=21 liposomes. *P<0.05; **P<0.01; ****P<0.0001. For P<0.0001, exact P values are not

available. Other *P* values are reported directly in the figure. Statistical significances, Kruskal-Wallis/Dunn's. For numerical source data, see **Supplementary Data 1**.

Supplementary Figure 2

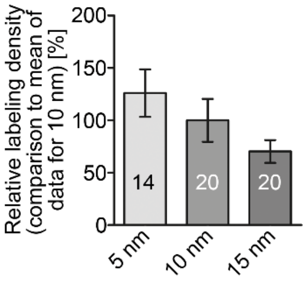

Supplementary Figure 2. Immunolabeling of freeze-fractured membranes show no strong dependence on the size of gold particles used for conjugation of secondary antibodies

Comparison of anti-PIP_2 immunogold labeling densities at cellular membranes obtained with 5 nm, 10 nm and 15 nm gold conjugates of secondary antibodies. Plasma membrane of NIH3T3 cells were used as they readily provide large areas of freeze-fractured membranes. Note that differences in labeling densities obtained with either 5 nm gold or 15 nm gold were modest when compared to the medium sized (10 nm) gold particles and that, despite the size differences of the probes, successful immunogold labelings were obtained in all three cases. 5 nm, n=14 images; 10 nm, n=20 images; 15 nm, n=20 images. Data, mean±SEM. Statistical significances, Kruskal-Wallis/Dunn's (all n.s.). For numerical source data, see **Supplementary Data 1**.

Supplementary Figure 3

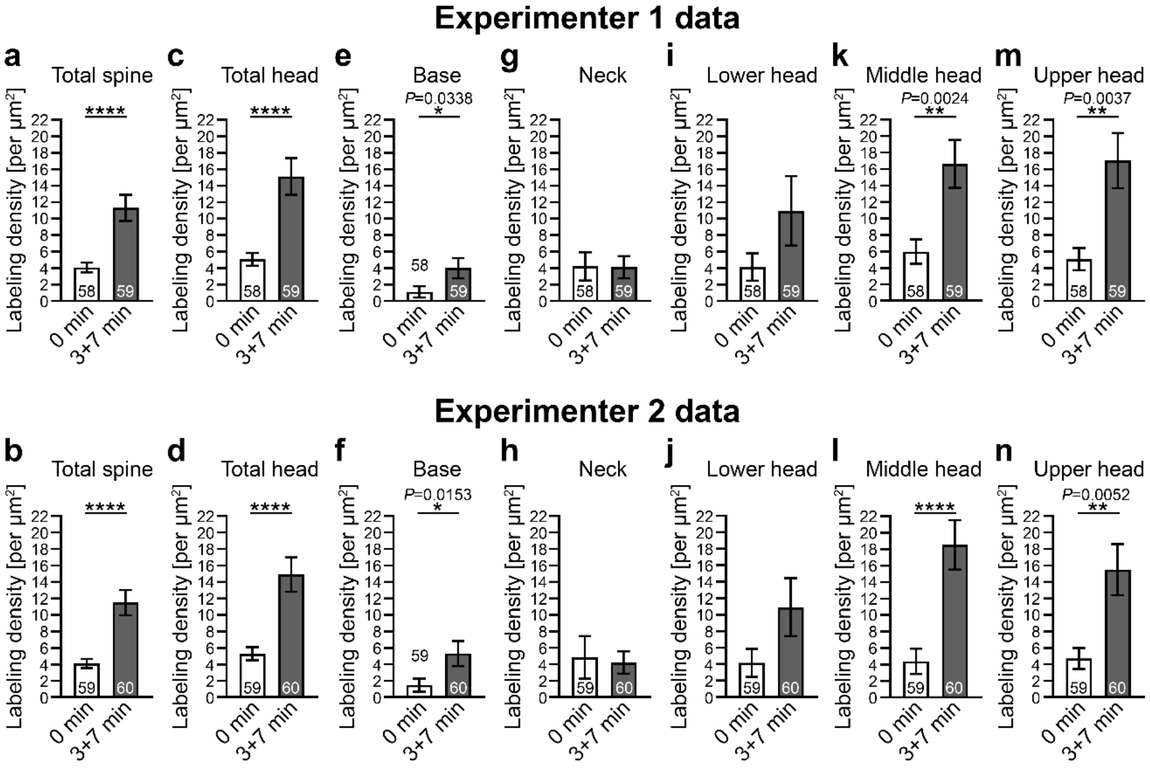

Supplementary Figure 3. Quantitative analyses of freeze-fractured primary hippocampal neurons subjected to NMDA-induced LTD by the original experimenter and independent evaluations conducted by a second and untrained experimenter lead to similar data sets

Comparison of data for 0 min and for 3+7 min NMDA stimulation (absolute data, i.e. not yet normalized to assay-intrinsic control and thus not shown as relative data) related to **Figure 3** and **Figure 4** data, which were obtained by the original experimenter (**a,c,e,g,i,k,m**) and by an untrained experimenter, respectively (**b,d,f,h,j,l,n**), simply following the evaluation protocol described in the Methods section. Note that the means of anti-PIP_2 immunogold labeling densities obtained by the independent examinations of the two experimenters for 0 min and for NMDA-induced LTD (3+7 min) in the total spine (**a,b**), in the total spine head (**c,d**), in the base area (**e,f**) and the spine neck area (**g,h**) as well as in the three subareas of the spine head (lower, middle and upper head; **i-n**) show virtually identical numbers. Note that minimal data deviations between the two experimenters in part result from the fact

that experimenter 2 evaluated 1 spine more in each of the two conditions (each condition contained 1 image with two spines). Detailed individual data comparisons demonstrate that the detection of labels, their assignment to membrane subareas in the spines as well as the size determinations of the spine subareas were not subjective but it was even possible for an untrained experimenter to reach virtually identical labeling density values for each area by following the pre-set protocol for base-setting, spine axis determination, head definition and head subdivision outlined in the Methods section. Experimenter 1, 0 min, n=58 images/spines; 3+7 min, 59 images/spines. Experimenter 2, 0 min, n=58 images/59 spines; 3+7 min, n=59 images/60 spines. 3+7 min images correspond to data shown in **Figure 3** and **Figure 4** in normalized form; 0 min images are corresponding control images plus 24 further control images to assemble a balanced data set for data comparisons conducted by two independent experimenters. Data, mean±SEM. Statistical significances, Mann-Whitney. *P<0.05; **P<0.01; ****P<0.0001. For P<0.0001, exact P values are not available. Other P values are reported directly in the figure. For numerical source data, see **Supplementary Data 1**.
