# Supplementary material for: Long-term depression in neurons involves temporal and ultra-structural dynamics of phosphatidylinositol-4,5-bisphosphate relying on PIP5K, PTEN and PLC
Source: Commun Biol. 2023 Apr 3;6:366. doi: 10.1038/s42003-023-04726-0 (PMC10070498; doi:10.1038/s42003-023-04726-0)
Supplement: Supplementary file 3 — Description of Additional Supplementary Files [file 42003_2023_4726_MOESM3_ESM.pdf]

Description of Additional Supplementary Files

File Name: Supplementary Data 1

Description: Compilation of all source data underlying the quantitative data presented as graphs in the paper.
