# Supplementary material for: Long-term depression in neurons involves temporal and ultra-structural dynamics of phosphatidylinositol-4,5-bisphosphate relying on PIP5K, PTEN and PLC
Source: Commun Biol. 2023 Apr 3;6:366. doi: 10.1038/s42003-023-04726-0 (PMC10070498; doi:10.1038/s42003-023-04726-0)
Supplement: Supplementary file 5 — Reporting Summary [file 42003_2023_4726_MOESM5_ESM.pdf]

nature portfolio

Corresponding author(s): Britta Qualmann & Michael M. Kessels

Last updated by author(s): January 24th, 2023

Reporting Summary

Nature Portfolio wishes to improve the reproducibility of the work that we publish. This form provides structure for consistency and transparency in reporting. For further information on Nature Portfolio policies, see our Editorial Policies and the Editorial Policy Checklist.

Statistics

For all statistical analyses, confirm that the following items are present in the figure legend, table legend, main text, or Methods section.

n/a	Confirmed	
☐	☒	The exact sample size (n) for each experimental group/condition, given as a discrete number and unit of measurement
☐	☒	A statement on whether measurements were taken from distinct samples or whether the same sample was measured repeatedly
☐	☒	The statistical test(s) used AND whether they are one- or two-sided<br>*Only common tests should be described solely by name; describe more complex techniques in the Methods section.*
☐	☒	A description of all covariates tested
☐	☒	A description of any assumptions or corrections, such as tests of normality and adjustment for multiple comparisons
☐	☒	A full description of the statistical parameters including central tendency (e.g. means) or other basic estimates (e.g. regression coefficient) AND variation (e.g. standard deviation) or associated estimates of uncertainty (e.g. confidence intervals)
☐	☒	For null hypothesis testing, the test statistic (e.g. F, t, r) with confidence intervals, effect sizes, degrees of freedom and P value noted<br>*Give P values as exact values whenever suitable.*
☐	☐	For Bayesian analysis, information on the choice of priors and Markov chain Monte Carlo settings
☐	☐	For hierarchical and complex designs, identification of the appropriate level for tests and full reporting of outcomes
☐	☐	Estimates of effect sizes (e.g. Cohen's d, Pearson's r), indicating how they were calculated

Our web collection on statistics for biologists contains articles on many of the points above.

Software and code

Policy information about availability of computer code

Data collection	only commercially available software or Freeware was used and all software used is listed in the manuscript
Data analysis	only commercially available software or Freeware was used and all software used is listed in the manuscript

For manuscripts utilizing custom algorithms or software that are central to the research but not yet described in published literature, software must be made available to editors and reviewers. We strongly encourage code deposition in a community repository (e.g. GitHub). See the Nature Portfolio guidelines for submitting code & software for further information.

Data

Policy information about availability of data

All manuscripts must include a data availability statement. This statement should provide the following information, where applicable:

- Accession codes, unique identifiers, or web links for publicly available datasets
- A description of any restrictions on data availability
- For clinical datasets or third party data, please ensure that the statement adheres to our policy

The authors declare that all data supporting the findings of this study are available within the paper and its supplementary information files.

Human research participants

Policy information about studies involving human research participants and Sex and Gender in Research.

Reporting on sex and gender	Does not apply
Population characteristics	*Describe the covariate-relevant population characteristics of the human research participants (e.g. age, genotypic information, past and current diagnosis and treatment categories). If you filled out the behavioural & social sciences study design questions and have nothing to add here, write "See above."*
Recruitment	*Describe how participants were recruited. Outline any potential self-selection bias or other biases that may be present and how these are likely to impact results.*
Ethics oversight	*Identify the organization(s) that approved the study protocol.*

Note that full information on the approval of the study protocol must also be provided in the manuscript.

Field-specific reporting

Please select the one below that is the best fit for your research. If you are not sure, read the appropriate sections before making your selection.

☒ Life sciences ☐ Behavioural & social sciences ☐ Ecological, evolutionary & environmental sciences

For a reference copy of the document with all sections, see nature.com/documents/nr-reporting-summary-flat.pdf

Life sciences study design

All studies must disclose on these points even when the disclosure is negative.

Sample size

No statistical methods were used to predetermine sample size.
All electron microscopical data obtained from freeze-fractured cells is usually based on n=30 - 63. Sampling was done in a systematic manner. Therefore, the n numbers merely reflect the outcome of the systematic searches and differ. Control samples (e.g. untreated spines of control cells) always examined in parallel to all conditions analyzed and therefore have higher n numbers (159 spine and correspondingly 159 neighboring dendritic areas).
When it comes to quatitative determinations of immunolabeling densities in EM experiments, the n numbers obtained from the 3-16 independent assays usually allow for a reliable visualization of increases to >150% of control by averaging. Such strong increases or the even higher ones observed in our study (200-300% of control) usually lead to the required statistical significances – provided that the membrane areas to be analyzed have a sufficient size and therefore compensate for the heterogeneity of labeling. The ability to quantitatively study labeling distribution and labeling intensity in perpendicular views onto the plasma membrane is a clear advantage of the applied FRIL/TEM method in comparison to classical sectioning techniques, which do not provide any perpendicular views onto membrane areas.

Data exclusions

No technically sound experimental data was excluded from the analyses reported. Also, no "outlier" analyses and exclusions were done.

Replication

There is no data in the manuscript that is merely shown in form of example pictures. Instead, all data in the different figures of the manuscript and in the supplementary figures are reported as quantitative analyses from multiple samples and independent assays.
N-numbers, numbers of independent assays and statistical significance analyses are reported directly in the figures and the legends, respectively.

Randomization

There was no allocation of samples into experimental groups except for the fact that certain rows in a cell culture plate were incubated with a given reagent and other rows with something else. As within one assay anyway all conditions were analyzed, i.e. no data/samples were excluded, and the cellular preparation is the same in all cell culture wells and several wells/coverslips were imaged for transfected cells, randomization of samples is not a concern in our study.

Blinding

All work with neurons was done in a fully blinded manner.
Blinding of samples was done by a colleage prior to imaging. Recorded images were blinded using the Ant Renamer Software, as described in Material and Methods.
Liposome work (Fig. 1a-e) was done by random imaging and subsequent determination of labeling densities of all liposomes found. Dito work for S2 (secondary antibody control examinations).

As demanded by the reviewers, the revised manuscript now also includes extended sets of EM images that have been independently evaluated by a different, untrained experimenter (additional experimenter 2). The work of experimenter 2 was not blinded but compared to the results of the (blinded) analyses conducted by experimenter 1.
See Supplementary Figure S3 for detailed comparisons of results shown in absolute values between the main experimenter (1) and the additional experimenter (2).
The results

Reporting for specific materials, systems and methods

We require information from authors about some types of materials, experimental systems and methods used in many studies. Here, indicate whether each material, system or method listed is relevant to your study. If you are not sure if a list item applies to your research, read the appropriate section before selecting a response.

Materials & experimental systems

n/a	Involved in the study
☐	☒ Antibodies
☒	☐ Eukaryotic cell lines
☒	☐ Palaeontology and archaeology
☐	☒ Animals and other organisms
☒	☐ Clinical data
☒	☐ Dual use research of concern

Methods

n/a	Involved in the study
☒	☐ ChIP-seq
☒	☐ Flow cytometry
☒	☐ MRI-based neuroimaging

Antibodies

Antibodies used	mouse monoclonal anti-PIP2 antibodies (Enzo Life Sciences) were used as primary antibodies.
Validation	For experimental validations of the anti-PIP2 antibody for the use in freeze-fracture immunolabeling and TEM please see Figure 1 of the manuscript and Supplementary Figures S1:<br>The specificity was quantitatively validated by using liposomes with either PIP2 added or PS added (also negatively charged lipid used as negative control) and with additional phosphoinositides added for further analyses of putative crossreactivities (Figure 1a-e). For individual data points compare Supplementary Figure S1.<br><br>The specificity of the established labeling procedures in neurons was furthermore validated by quantitative determinations of the labeling in the P-face of the membrane vs. the extracellularly oriented E-face and ice surfaces as intrinsic controls in the same samples in soma (Figure 1f,g) and in dendritic plasma membrane areas (Figure 1h-k).<br>The specificity of the established labeling procedures in neurons was furthermore validated by quenching the primary antibody and quantitative determinations of the remaining labeling in the P-face (Figure 1i and k).<br><br>Saturation of labeling was determined by quantitative labeling examinations of dendrites of primary hippocampal neurons using three differerent concentrations of the antibody (Figure 1l-o).<br><br>Putative dependences of secondary antibody-gold conjugates on the sizes of the colloidal gold particles used were assessed in additional quantitative determinations of labeling densities (Supplementary Figure S2).

Animals and other research organisms

Policy information about studies involving animals; ARRIVE guidelines recommended for reporting animal research, and Sex and Gender in Research

Laboratory animals	Rats (Crl:WI; Charles River) were used for primary cell culture preparations only.
Wild animals	none
Reporting on sex	mixed (ratio undetermined), as embryos were used and the hippocampi of all embryos of one mother animal were pooled for the generation of each primary cell culture.
Field-collected samples	none
Ethics oversight	No specific ethical oversight required. Tissue material use from sacrificed animals only.

Note that full information on the approval of the study protocol must also be provided in the manuscript.
